# Influence of preoperative embolisation on resection of brain arteriovenous malformations: cohort study

**DOI:** 10.1007/s00701-024-06234-4

**Published:** 2024-08-21

**Authors:** Seong Hoon Lee, James JM. Loan, Jonathan Downer, Johannes DuPlessis, Peter Keston, Anthony N. Wiggins, Ioannis Fouyas, Drahoslav Sokol

**Affiliations:** 1 Department of Clinical Neurosciences, Royal Infirmary of Edinburgh, Edinburgh, UK; 2 South West Neurosurgery Centre, Derriford Hospital, Plymouth, UK; 3 Centre for Clinical Brain Sciences, University of Edinburgh, Edinburgh, UK

**Keywords:** Arteriovenous malformations, Intracranial arteriovenous malformations, AVM, Embolization, Preoperative embolization, Cerebrovascular procedures

## Abstract

**Purpose:**

Preoperative endovascular embolisation is a widely used adjunct for the surgical treatment of brain arteriovenous malformations (AVMs). However, whether this improves completeness of AVM resection is unknown, as previous analyses have not adjusted for potential confounding factors. We aimed to determine if preoperative endovascular embolisation was associated with increased rate of complete AVM resection at first surgery, following adjustment for Spetzler-Martin grade items.

**Methods:**

We identified a cohort of all patients undergoing first ever AVM resection in a specialist neurosciences unit in the NHS Lothian Health Board region of Scotland between June 2004 and June 2022. Data was prospectively extracted from medical records. Our primary outcome was completeness of AVM resection. We determined the odds of complete AVM resection using binomial logistic regression with adjustment for Spetzler-Martin grading system items: maximum nidus diameter, eloquence of adjacent brain and the presence of deep venous drainage.

**Results:**

88 patients (median age 40y [IQR 19–53], 55% male) underwent AVM resection. 34/88 (39%) patients underwent preoperative embolisation and complete resection was achieved at first surgery in 74/88 (84%). Preoperative embolisation was associated with increased adjusted odds of complete AVM resection (adjusted odds ratio [aOR] 8.6 [95% confidence interval (95% CI) 1.7–67.7]; *p* = 0.017). The presence of deep venous drainage was associated with reduced chance of complete AVM resection (aOR 0.18 [95% CI 0.04–0.63]; *p* = 0.009).

**Conclusions:**

Preoperative embolisation is associated with improved chances of complete AVM resection following adjustment for Spetzler-Martin grade, and should therefore be considered when planning surgical resection of AVMs.

**Supplementary Information:**

The online version contains supplementary material available at 10.1007/s00701-024-06234-4.

## Introduction

Brain arteriovenous malformations (AVMs) are intraparenchymal vascular lesions, composed of an arteriovenous shunt without an intervening capillary bed. AVMs can lead to substantial neurological disability or mortality, most commonly through intracranial haemorrhage [2, 14]. To reduce this risk, surgical treatment aims for complete obliteration of the AVM [4, 8].

Preoperative endovascular embolisation is a widely used adjunct for the surgical excision of AVMs, with the goal of reducing operative risk. Successful embolisation reduces nidal blood flow, AVM size, and intra-operative bleeding [14]. However, embolisation also carries potential risks, including ischaemic stroke, haemorrhage from altered lesion flow dynamics or technical procedural complications [18]. Although these risks and benefits are well recognised, a recent systematic review found very limited evidence on the impact of preoperative AVM embolisation in improving completeness of AVM resection [14]. To date, no randomised controlled trials have been conducted to address this question and no observational analysis of the association of pre-operative embolisation with completeness of AVM resection, which adjust for potential confounding factors have been published [14]. The lack of adjusted analyses is important, as in observational studies, patients are selected for pre-operative embolisation based on their clinical characteristics. These are encompassed by the Spetzler-Martin score, which is a highly validated measure of operative risk for brain AVM surgery [9]. Unadjusted rates of completeness of resection are therefore of limited utility in assessing the role of preoperative embolisation, as patients undergoing preoperative embolisation are typically of higher Spetzler-Martin grade than those that do not [14].

Because the main aim of AVM surgery is complete removal of the AVM, the lack of published analyses on completeness of AVM resection that account for pre-operative differences between patients who do or do not undergo preoperative embolisation is a critical knowledge gap. To address this, we aimed to determine whether preoperative embolisation was associated with increased completeness of AVM resection, following adjustment for Spetzler-Martin score components: nidus size, location and the presence of deep venous drainage.

## Methods

This study has been reported in accordance with the STROBE guideline for cohort studies [21].

### Study design and participants

We performed a cohort study including all consecutive patients undergoing first ever AVM resection in a specialist neurosciences unit of a university teaching hospital in the NHS Lothian Health Board of Scotland between June 2004 and June 2022. Patients were included if they underwent first ever surgical resection of a brain arteriovenous malformation during the study period, irrespective of previous non-surgical treatment or clinical status. Those undergoing redo surgery were not excluded.

### Interventions

All patients underwent craniotomy and open microsurgical excision of their AVM. Surgical approach and use of operative adjuncts including neuronavigation and intraoperative indocyanine green angiography were at the discretion of the operating consultant neurosurgeon. Preoperative embolisation was performed by a consultant interventional neuroradiologist following multidisciplinary team discussion to determine the treatment goal. This was typically to occlude deep arterial feeders which were predicted to be difficult to immediately control during surgery, and reduce nidal size, rather than complete AVM occlusion. Biplanar digital subtraction angiography (DSA) was used to guide dimethyl sulphoxide compatible microcatheters to the target vessel for transarterial embolisation. Following initial angiographic runs to confirm their contribution to the AVM and safety of runoff, the chosen embolic agent was cautiously injected under angiographic control to selectively occlude the target vessel and safely penetrate nidus. The selection and concentration of embolic agent, mostly ethylene–vinyl alcohol copolymer (Onyx) or precipitating hydrophobic injectable liquid (PHIL) was at the discretion of the treating radiologist (Supplementary Table 1). Post-embolisation DSA runs were immediately performed to confirm target vessel occlusion, nidal reduction and appropriate patency of venous drainage and vessels supplying other critical structures.

### Baseline and outcome variables

Consecutive patients were prospectively ascertained by the operating consultant neurosurgeons (IF, DS). We extracted anonymised clinical and radiological data from medical records and imaging systems. Data on baseline patient demographics, anti-thrombotic usage/coagulopathy, and presenting symptoms were collected. Radiographic data on AVM location, Spetzler-Martin grade (maximum nidus diameter, eloquence of adjacent brain, deep venous drainage), as well as Spetzler-Martin-Lawton extended grade (Spetzler-Martin grading items plus age at resection, haemorrhage prior to resection, compactness) were extracted from consultant neuroradiologist imaging reports (JD, JDP, PK). Data points not specifically addressed in imaging reports were verified by a neurosurgical clinical lecturer (JJML).

Embolisation and operative data on embolic material used, duration between preoperative embolisation and surgery, and duration of surgery were collected. Post-operative radiological completeness of resection was assessed (as ascertained on DSA by a multidisciplinary team of consultant neuroradiologists and neurosurgeons), as were post-operative clinical outcomes on duration of inpatient stay, ICU admission, neurological function (modified Rankin scale [mRS]), postoperative complications (haemorrhage, focal neurological deficit, surgical/wound infection, hydrocephalus, seizures, and acute cardiovascular/thrombotic events), and mortality on discharge.

### Bias and study size

All patients undergoing first ever AVM resection were consecutively recruited to minimise selection bias. A comprehensive set of variables allowed for the control of confounders. There is no consensus on the method of power calculation for multivariable logistic regression and so the maximal available sample size was used [19].

### Statistical analysis

Our primary outcome was the adjusted odds of completeness of AVM resection associated with preoperative embolisation in an intention to treat analysis. This was determined using binomial logistic regression with adjustment for Spetzler-Martin grading components [17]. We included data from all available patients and those with missing data were dropped per analysis. To adjust for Spetzler-Martin grade, we considered eloquence and the presence of deep venous drainage as binomial categorical variables. Maximum nidus diameter was a continuous variable. Additionally, we performed an exploratory analysis using the Spetzler-Martin-Lawton-Young extended grading scale components with the presence of haemorrhage and nidus compactness as binomial categorical variables and patient age as continuous [11]. We performed further exploratory analyses by stratifying according to the presence of deep venous drainage and by adding an additional covariable to adjust for surgery being undertaken on an emergency basis (< 12 h from symptom onset), versus an urgent (< 2 weeks) or semi-elective (> 2 weeks) basis. Over our 18-year study period it was possible that overall secular trends in patient care or non-secular variation affecting epochs of care might have influenced results. To account for possible secular trends, we used an exploratory model of our primary outcome including the order in which patients underwent surgery over the whole cohort as a covariate. To account for non-secular variation, we repeated our main analysis of completeness of resection adjusted for Spetzler-Martin grade items in a mixed-effects logistic regression with a random intercept of order of treatment. In case infratentorial location influenced outcome, we performed an exploratory analysis with adjustment for infratentorial location in place of the Spetzler-Martin grade item of eloquent location. Secondary outcomes including death or dependency at discharge (mRS 3–6), post-operative intensive care unit admission, mortality, duration of admission, and duration of surgery were analysed using binomial logistic and linear regression models for categorical and continuous variables, respectively. For linear regression, all dependent variables were found to be skewed and as such were log-transformed to achieve normalisation prior to analysis.

Univariable analyses of categorical variables were performed using the Wilcoxon rank sum test, Pearson’s Chi-squared test, or Fisher’s exact test. Using 2-sided *P*-values, statistical significance was set at *P* < 0.05. Statistical analysis was performed on R Project (R Core Team [2022]. R Foundation for Statistical Computing, Vienna, Austria).

## Results

### Patient characteristics

This study included 88 patients (median age 40 years; inter-quartile range [IQR] 19–53, 54.5% males) undergoing first ever AVM resection. Of these patients, 38.6% (*n*/*N* = 34/88) received preoperative embolisation and 61.4% (*n*/*N* = 54/88) underwent surgical resection alone. The median mRS at admission was 2 for patients treated both with and without preoperative embolisation. A greater proportion of patients who received surgery without preoperative embolisation were dependent (mRS > 2) at admission (42.6%; *n*/*N* = 23/54), compared with those who received preoperative embolisation (20.6%; *n*/*N* = 7/34). The majority of patients demonstrated symptoms attributable to their AVMs, with headache, focal neurological deficit, and reduced levels of consciousness being the commonest presenting symptoms. Haemorrhage and perceived haemorrhage risk were the commonest indications for intervention (Table 1).

**Table 1:** Patient characteristics

Variables: Median [IQR]; *n* (%)	Total (*N* = 88)	Surgery only (*N *= 54)	Preoperative embolisation (*N* = 34)	*P*-value
Age	40 [19 – 53]	39 [20 – 54]	41 [18 – 52]	> 0.9
Female	40 (46%)	26 (48%)	14 (41%)	0.5
mRS at admission				0.09
0	9 (10%)	7 (13%)	2 (5.9%)	
1	28 (32%)	15 (28%)	13 (38%)	
2	21 (24%)	9 (17%)	12 (35%)	
3	12 (14%)	8 (15%)	4 (12%)	
4	3 (4%)	2 (4%)	1 (3%)	
5	15 (17%)	13 (24%)	2 (6%)	
Dependency (mRS > 2) at admission	30 (34%)	23 (43%)	7 (21%)	0.034
Presenting symptoms				0.2
Headache	28 (32%)	17 (31%)	11 (32%)	
Focal neurological deficit	24 (27%)	16 (30%)	8 (24%)	
Reduced consciousness	19 (22%)	14 (26%)	5 (15%)	
Seizure	12 (14%)	4 (7%)	8 (24%)	
Unknown	5 (6%)	3 (6%)	2 (6%)	
Indication for intervention				0.7
Haemorrhage	28 (32%)	19 (35%)	9 (26%)	
Risk of haemorrhage	12 (14%)	7 (13%)	5 (15%)	
Seizures	5 (6%)	2 (4%)	3 (9%)	
Focal neurological deficit	1 (1%)	1 (2%)	0 (0%)	
Multiple of above	42 (48%)	22 (41%)	17 (50%)	0.2

Most AVMs were of supratentorial lobar location and the median nidus maximum diameter was 2.0 cm (IQR 1.3 – 3.2; Table 2). 39% (*n*/*N* = 34/88) of AVMs were located in eloquent regions; with 38% (*n*/*N* = 33/88) located in the frontal, 18% (*n*/*N* = 16/88) parietal, and 15% (*n*/*N* = 13/88) temporal lobes. One eloquent AVM was in the corpus callosum. 42% (*n*/*N* = 37/88) of AVMs had deep venous drainage, with a greater proportion of preoperative embolisation patients having high Spetzler-Martin grades compared to those treated by surgery only (Table 2).

**Table 2:** AVM characteristics

Variables: Median [IQR]; *n* (%)	Total (*N* = 88)	Surgery only (*N* = 54)	Preoperative embolisation (*N* = 34)	*P*-value
AVM location				0.4
Frontal	33 (38%)	21 (39%)	12 (35%)	
Cerebellar	19 (22%)	11(20%)	8 (24%)	
Parietal	16 (18%)	10 (19%)	6 (18%)	
Temporal	13 (15%)	10 (19%)	3 (8.8%)	
Occipital	6 (7%)	2 (4%)	4 (12%)	
Callosal	1 (1%)	0 (0%)	1 (3%)	
Nidus maximum diameter (cm)	2.0 [1.3 – 3.2]	1.7 [1.2 – 2.1]	3.0 [2.0 – 4.0]	< 0.001
Deep venous drainage	37 (42%)	16 (30%)	21 (62%)	0.003
Eloquent location	34 (39%)	18 (33%)	16 (47%)	0.2
Haemorrhage	67 (76%)	43 (80%)	24 (71%)	0.3
Compactness	58 (66%)	35 (65%)	23 (68%)	0.8
Spetzler-Martin Grade				0.002
1	41 (47%)	32 (59%)	9 (26%)	
2	25 (28%)	15 (28%)	10 (29%)	
3	22 (25%)	7 (13%)	15 (44%)	
4	0 (0%)	0 (0%)	0 (0%)	
5	0 (0%)	0 (0%)	0 (0%)	
Spetzler-Martin Extended Grade				0.043
1	0 (0%)	0 (0%)	0 (0%)	
2	10 (11%)	9 (17%)	1 (3%)	
3	19 (22%)	13 (24%)	6 (18%)	
4	30 (34%)	17 (31%)	13 (38%)	
5	20 (23%)	13 (24%)	7 (21%)	
6	9 (10%)	2 (4%)	7 (21%)	

### Treatment

58% (n/N = 51/88) of patients received treatment semi-electively, 23% (n/N = 20/88) urgently and 19% (n/N = 17/88) on an emergency basis (Table 3). Patients who received preoperative embolisation were more likely to be managed on a semi-elective basis compared with those who did not receive embolisation (Table 3). In comparison to patients treated with surgical resection alone (median Spetzler-Martin grade 1; [IQR 1 – 2]), a greater proportion of patients treated with preoperative embolisation (median Spetzler-Martin grade 2; [IQR 1 – 3]) were of higher Spetzler-Martin grade (*P* = 0.002), with greater nidus diameters and more frequent deep venous drainage (Table 2).

**Table 3:** Embolisation and surgery outcomes

Variables Median [IQR]; n (%)	Total (N = 88)	Surgery only (N = 54)^1^	Preoperative embolisation (N = 34)^1^	*P*-value^2^
Urgency of intervention				0.02
Semi-elective	51 (58%)	26 (48%)	25 (74%)	
Urgent	20 (23%)	13 (24%)	7 (21%)	
Emergency	17 (19%)	15 (28%)	2 (6%)	
Completeness of embolisation				< 0.001
Partial		NA	31 (91%)	
Not embolised	57 (65%)	54 (100%)	3 (9%)	
Completeness of resection				0.04
Incomplete	14 (16%)	12 (22%)	2 (6%)	
Complete	74 (84%)	42 (78%)	32 (94%)	
Embolisation complications				0.15
Extracranial vessel injury		NA	1 (3%)	
None		NA	33 (97%)	
Time from embolisation to surgery (days)		NA	0 [0–7]	
Duration of surgery (min)	185 [115 – 269]	190 [123 – 237]	150 [110 – 335]	0.9
Second look surgery required	9 (10%)	8 (15%)	1 (3%)	0.1
Postoperative complications	24 (27%)	14 (26%)	10 (29%)	0.7
mRS at discharge				0.4
0	13 (15%)	9 (17%)	4 (12%)	
1	31 (35%)	15 (28%)	16 (47%)	
2	18 (20%)	14 (26%)	4 (12%)	
3	17 (19%)	9 (17%)	8 (24%)	
4	3 (4%)	3 (5.6%)	0 (0%)	
5	1 (1%)	1 (2%)	0 (0%)	
6 (mortality)	4 (5%)	3 (6%)	1 (3%)	> 0.9
Duration of inpatient admission (days)	12 [7 – 23]	12 [7 – 23]	9 [7 - 22]	0.4
Intensive Care Unit admission	32 (36%)	23 (43%)	9 (26%)	0.1
Duration of Intensive Care Unit admission (days)	0 [0 – 2]	0 [0 – 5]	0 [0 – 1]	0.09

Among patients undergoing attempted preoperative embolisation, 91% (*n/N* = 31/34) had partial embolisation of their AVMs and 9% (*n/N* = 3/34) could not be embolised. The median time between embolisation to surgical resection was 0 days [IQR 0 – 7]. Complete resection was achieved at first surgery in 78% (*n/N* = 42/54) of surgery only patients and in 94% (n/N = 32/34) for preoperative embolisation patients (*P* = 0.041; Table 3). The median duration of surgery for surgery only patients was 190 min [IQR 123 – 237], versus 150 min [IQR 110 – 335] for preoperative embolisation patients (*P* = 0.9; Table 3). Eight surgery only patients required a second operation due to incomplete initial resection, compared to just one for the preoperative embolisation cohort (*P* = 0.14).

There were three inpatient deaths among surgery only patients, whom all presented as comatose (GCS < 8 on admission) and subsequently underwent emergency surgery. One patient who underwent preoperative embolisation subsequently died of a pulmonary embolism. Specific complications and embolic materials used are detailed in Supplementary Table 1.

### Adjusted analysis of resection completeness

In our primary analysis, preoperative embolisation was associated with increased odds of complete AVM resection (adjusted odds ratio [aOR] 8.6; 95% confidence interval [95% CI] 1.7–67.7; p = 0.017), following adjustment for Spetzler-Martin criteria. In this model, the presence of deep venous drainage was associated with reduced odds of complete AVM resection (aOR 0.18; 95% CI 0.04–0.63; *p* = 0.009). In an exploratory analysis with inclusion of an additional covariable to adjust for surgery undertaken on an emergency basis, the association between embolisation and complete AVM resection remained (aOR 6.7; 95% CI 1.2 – 55.3; *p* = 0.042). A sensitivity analysis indicated that this association was particularly marked for AVM patients with deep venous drainage (aOR 14.6; 95% CI 1.9–205.2; *p* = 0.02; Table 4). In further exploratory analyses we found no evidence that this association was influenced by temporal trends or variations in care across the study period (Supplementary Table 2).

**Table 4:** Binomial logistic regression analyses of association of embolisation with complete AVM resection adjusted for Spetzler-Martin grade components. *95% Confidence interval

Variable	aOR (95% CI*)	*P*-value
Prespecified model including all patients		
Embolisation	8.6 (1.7 – 67)	0.017
Eloquent location	1.0 (0.3–4.1)	> 0.9
Nidus diameter (per cm)	1.0 (0.7–1.9)	> 0.9
Deep venous drainage	0.18 (0.04–0.6)	0.01
Sensitivity analysis of only patients with deep venous drainage		
Embolisation	14 (1.9–205)	0.021
Eloquent location	0.7 (0.13–4.4)	0.7
Nidus diameter (per cm)	0.62 (0.24–1.5)	0.3
Exploratory analysis including association with emergency treatment		
Embolisation	6.7 (1.2–55)	0.042
Eloquent location	0.93 (0.23–4.0)	> 0.9
Nidus diameter (per cm)	1.0 (0.63–1.8)	> 0.9
Deep venous drainage	0.14 (0.03–0.55)	0.007
Emergency surgery	0.23 (0.05–0.96)	0.046

We also performed an exploratory analysis with adjustment for the Spetzler-Martin-Lawton-Young extended grading system items. In this analysis, the associations of embolisation and deep drainage remained consistent, but age, nidus, compactness, and the presence of haemorrhage were not associated with resection completeness (Supplementary Table 3). Similarly, adjustment for infratentorial location of AVMs (in place of the Spetzler-Martin item of eloquent location), did not influence the observed adjusted association of embolisation with resection completeness (Supplementary Table 4).

We did not find significant associations between embolisation status and secondary outcome measures of death or dependency at discharge (mRS 3–6), inpatient mortality, post-operative intensive care unit admission, duration of admission, or duration of surgery (Supplementary Tables 5–8).

## Discussion

In a cohort of 88 patients undergoing first-ever AVM resection, we found that preoperative embolisation was associated with increased odds of complete AVM resection following adjustment for nidus diameter, presence of deep venous drainage, and eloquence of surrounding brain tissue. An exploratory sensitivity analysis indicated that the benefits of preoperative embolisation may be particularly pronounced for patients with deep venous drainage. However, this analysis was conducted in a small subgroup of patients and resulted in wide confidence intervals around the estimates of association. As such, caution should be employed in their interpretation of these exploratory findings, which would benefit from independent validation.

These results are in keeping with aggregated data from a recent meta-analysis; preoperative embolisation reduced AVM lesion volumes and was associated with excellent complete resection rates (96.6%; 95% CI 95.4 – 97.9) [14]. We demonstrate comparable complete resection and mortality rates of 94% and 2.9%, respectively. In a study of patients with exclusively low grade AVMs (Spetzler-Martin grades I-II), complete resection rates of 94% were achieved with 43% of their patients undergoing preoperative embolisation [16]. Other multi-centre studies involving Spetzler-Martin grade III-V AVMs report complete resection rates of 82% and overall mortality rates of 3.6% [1]. Our cohort size of 88 patients is in the highest third of studies of embolisation-assisted AVM resection included in a 2022 systematic review [14].

Although Spetzler-Martin characteristics are known to influence operative risk and treatment outcome, there are no studies which investigate the association of embolisation with completeness of AVM resection that adjust for these variables [9]. Although residual confounding arising from more complex AVMs being more likely to receive embolisation may be present in our analysis, this would be expected to reduce the magnitude of the adjusted association of embolisation with complete resection which we identified and therefore we think this is unlikely to have influenced our conclusions. Nonetheless, our work would therefore benefit from independent confirmatory studies. Definitive investigation of whether embolisation improves completeness of AVM resection requires a randomised clinical trial. To justify and guide such an endeavour however, adjusted observational analyses are required.

There are few studies on the role of preoperative embolisation in AVM surgery with regards to other outcomes that adjust for potential confounders. These studies emphasise the nuances of AVM management and the need for individualised treatment but provide varying results. A study providing propensity-adjusted analysis of patients with grade III AVMs showed that embolised patients displayed less post-operative dependency (mRS < 3) [4]. Contrastingly, Donzelli et al*.* demonstrate that preoperative embolisation was associated with longer median resection times, with no influence on intraoperative blood loss or mRS change post-operatively [7]. In an unadjusted case–control study, Luksik et al*.* found no association between preoperative embolisation and AVM obliteration or post-operative mRS [12]. Our study also demonstrated no significant associations between preoperative embolisation and in-hospital mortality or dependency at discharge. Aggregated data from previous meta-analyses support its overall safety profile [5, 10, 13, 15, 20].

Timing of surgery may impact on outcomes and some studies suggest that delayed surgery may reduce the risk of worsening neurological deficits following AVM resection and may also be associated with increased likelihood of complete resection [3, 6, 22]. It is also possible that the goals of surgery undertaken in an emergent fashion might differ from that done in a more planned manner; in emergency surgery haematoma evacuation may be prioritised before complete resection of AVMs. Further, in our study, we found that preoperative embolisation was undertaken more frequently for AVMs undergoing resection in a non-emergent fashion. To account for this potential confounder, we performed an exploratory analysis with ‘emergency surgery’ as an added covariate. The association of preoperative embolisation with improved completeness of resection remained, indicating that these associations were not solely due to confounding from surgical urgency.

Limitations of this cohort study include a potential selection bias. AVM patients were recruited by the treating consultant neurosurgeon/neuroradiologist who deemed the patient suitable for surgical resection and/or preoperative embolisation. However, by including consecutively treated patients we mitigated against selection biases to widen the generalisability of our cohort. One might expect that selection for embolisation would favour more challenging cases in receiving embolisation, and thus less complete resection. Although patients undergoing embolisation demonstrated higher Spetzler-Martin grades, resection remained more complete in this group, suggesting that embolisation was indeed effective. It is difficult to directly account for multifaceted developments in surgical and neurointerventional techniques which occurred over the study period and this is a potential limitation. Nonetheless we did not find that the association which we report was influenced by the point at which patients underwent treatment, and so we think this is unlikely to have significantly influenced our conclusions. AVMs are an uncommon condition and most published analyses related to their embolisation include fewer than 88 patients; our sample size would place it in the top 33% of studies included in a systematic review of associations between embolisation and outcome following AVM surgery (14). Nonetheless, we were potentially limited by our sample size of 88 patients to detect associations with certain covariables with our study outcomes. For example, the lack of detected adjusted associations between eloquent location and completeness of resection in our study may therefore reflect a type 2 error. Whilst we think that it is unlikely that our positive findings reflect a low sample size, it is important that readers consider our study population when assessing generalisability to other patient cohorts. Our study had no patients with Spetzler-Martin grade IV or V AVMs and so our findings cannot be generalised to this population.

## Conclusions

In a single centre study of 88 patients, preoperative embolisation was associated with greater chance of complete resection of brain AVMs, following adjustment for Spetzler-Martin grade. This association was particularly pronounced for AVMs with deep venous drainage. Pre-operative embolisation may therefore provide a valuable surgical adjunct in achieving complete AVM resection at first surgery. Independent confirmatory studies and meta-analyses which adjust for potential confounders are warranted, to assess the generalisability of our findings and to justify and guide the design of future definitive randomised clinical trials.

## Supplementary Information

Below is the link to the electronic supplementary material.

Supplementary file1 (DOCX 31 KB)

## Data Availability

Access to data may be provided to researchers with appropriate approvals by contacting the corresponding author.

## Code availability

Scripts for data analysis can be provided by contacting the corresponding author.
