# Correction to: Influence of preoperative embolisation on resection of brain arteriovenous malformations: cohort study

**DOI:** 10.1007/s00701-024-06273-x

**Published:** 2024-09-21

**Authors:** Seong Hoon Lee, James JM. Loan, Jonathan Downer, Johannes DuPlessis, Peter Keston, Anthony N. Wiggins, Ioannis Fouyas, Drahoslav Sokol

**Affiliations:** 1 Department of Clinical Neurosciences, Royal Infirmary of Edinburgh, Edinburgh, UK; 2 South West Neurosurgery Centre, Derriford Hospital, Plymouth, UK; 3 Centre for Clinical Brain Sciences, University of Edinburgh, Edinburgh, UK

**Correction to** **:** **Acta Neurochirurgica (2024)**

10.1007/s00701-024-06234-4

The affiliation of the last author Drahoslav Sokol is missing in the original paper. His affiliation is:

Department of Clinical Neurosciences, Royal Infirmary of Edinburgh, Edinburgh, UK.

The original article has been corrected.

The Publisher regrets this error.

