# Supplementary material for: Influence of preoperative embolisation on resection of brain arteriovenous malformations: cohort study
Source: Acta Neurochir (Wien). 2024 Aug 21;166(1):345. doi: 10.1007/s00701-024-06234-4 (PMC11339179; doi:10.1007/s00701-024-06234-4)
Supplement: Supplementary file 1 — Supplementary file1 (DOCX 31 KB) [file 701_2024_6234_MOESM1_ESM.docx]

**Supplementary Table 1:** Embolisation material used and post-surgical complications.

| **Variables** | **Total**  **(N = 88)** | **Surgery only**  **(N = 54)** | **Preoperative embolisation**  **(N = 34)** | ***P*-value** |
| --- | --- | --- | --- | --- |
| **Embolisation material** |  |  |  | >0.9 |
| PHIL |  | NA | 17 (50%) |  |
| Onyx |  | NA | 11 (32%) |  |
| Other |  | NA | 2 (6%) |  |
| Unknown |  | NA | 4 (12%) |  |
| **Surgery complications** |  |  |  | >0.9 |
| Delayed haematoma (> 24hr post-op) |  | 1 (2%) | 0 (0%) |  |
| Early haematoma (< 24hr post-op) |  | 1 (2%) | 0 (0%) |  |
| Focal neurological deficit |  | 4 (7%) | 3 (9%) |  |
| Infection |  | 0 (0%) | 1 (3%) |  |
| New hydrocephalus |  | 1 (2%) | 0 (0%) |  |
| Other |  | 3 (6%) | 2 (6%) |  |
| Seizures |  | 3 (6%) | 1 (3%) |  |
| Multiple of above |  | 1 (2%) | 2 (6%) |  |
| No documented complications |  | 40 (74%) | 25 (74%) |  |
| **Post-operative haemorrhage from AVM during study period** | 1 (1.1%) | 1 (1.9%) | 0 (0%) | >0.9 |
|  | | | | |

**Supplementary Table 2:** Binomial logistic regression analyses of association of embolisation with completeness of resection adjusted for Spetzler-Martin grade components and order of surgery from first resection performed in series to last using fixed and mixed effects models. aOR Adjusted odds ratio; *95% Confidence interval.

| **Variable** | **aOR (95% CI*)** | **P-value** |
| --- | --- | --- |
| *Fixed effects* |  |  |
| Embolisation | 8.2 (1.6 – 66.7) | 0.22 |
| Eloquent location | 1.3 (0.32 – 5.9) | 0.73 |
| Nidus diameter (per cm) | 0.96 (0.59 – 1.8) | 0.88 |
| Deep venous drainage | 0.17 (0.04 – 0.61) | 0.0087 |
| Order of procedure | 1.0 (0.99 – 1.0) | 0.24 |
| *Mixed effects* |  |  |
| **Random intercept** | **Standard Deviation** | **P-value** |
| Order of procedure | 0.25 |  |
| **Fixed effects** | **aOR (95% CI*)** | **P-value** |
| Embolisation | 8.6 (1.5 – 25.6) | 0.18 |
| Eloquent location | 1.0 (0.26 – 3.8) | >0.9 |
| Nidus diameter (per cm) | 1.0 (0.61 – 1.7) | >0.9 |
| Deep venous drainage | 0.18 (0.046 – 0.66) | 0.01 |

**Supplementary Table 3:** Binomial logistic regression analyses of association of embolisation completeness of resection adjusted for Spetzler-Martin-Lawton-Young extended grade components. aOR Adjusted odds ratio; *95% Confidence interval.

| **Variable** | **aOR (95% CI*)** | **P-value** |
| --- | --- | --- |
| Embolisation | 10.2 (1.7 – 96.0) | 0.020 |
| Eloquent location | 1.1 (0.28 – 4.6) | 0.90 |
| Nidus diameter (per cm) | 0.96 (0.59 – 1.8) | 0.88 |
| Deep venous drainage | 0.15 (0.03 – 0.59) | 0.0096 |
| Age (per year) | 1.02 (0.988 – 1.1) | 0.24 |
| Previous haemorrhage | 0.23 (0.01 – 1.5) | 0.20 |
| Nidus compactness | 0.70 (0.13 – 3.1) | 0.67 |

**Supplementary Table 4:** Binomial logistic regression analyses of association of embolisation completeness of resection adjusted for infratentorial location, nidus diameter and the presence of deep venous drainage. aOR Adjusted odds ratio; *95% Confidence interval.

| **Variable** | **aOR (95% CI*)** | **P-value** |
| --- | --- | --- |
| Embolisation | 7.5 (1.5 – 59.2) | 0.028 |
| Infratentorial location | 3.9 (0.6 – 77.0) | 0.22 |
| Nidus diameter (per cm) | 1.1 (0.69 – 2.0) | 0.78 |
| Deep venous drainage | 0.18 (0.04 – 0.66) | 0.012 |

**Supplementary Table 5:** Binomial logistic regression analyses of association of embolisation with postoperative ICU admission adjusted for Spetzler-Martin grade components. aOR Adjusted odds ratio; NA Not calculable due to complete separation; *95% Confidence interval.

| **Variable** | **aOR (95% CI*)** | **P-value** |
| --- | --- | --- |
| *Prespecified model including all patients* | | |
| Embolisation | 0.45 (0.15 – 1.3) | 0.14 |
| Eloquent location | 0.72 (0.27 – 1.8) | 0.49 |
| Nidus diameter (per cm) | 1.2 (0.86 – 1.69) | 0.30 |
| Deep venous drainage | 0.72 (0.27 – 1.92) | 0.52 |
| *Sensitivity analysis of only patients with deep venous drainage* | | |
| Embolisation | 0.40 (0.06 – 2.2) | 0.30 |
| Eloquent location | 1.4 (0.31 – 6.4) | 0.64 |
| Nidus diameter (per cm) | 1.1 (0.54 – 2.4) | 0.73 |
| *Exploratory analysis including association with emergency treatment* | | |
| Embolisation | 0.77 (0.20-2.8) | 0.69 |
| Eloquent location | 0.78 (0.22-2.5) | 0.68 |
| Nidus diameter (per cm) | 1.3 (0.89-2.0) | 0.17 |
| Deep venous drainage | 0.87 (0.25-2.9) | 0.82 |
| Emergency surgery | NA | >0.9 |

**Supplementary Table 6:** Binomial logistic regression analyses of association of embolisation with postoperative complications adjusted for Spetzler-Martin grade components. aOR Adjusted odds ratio; *95% Confidence interval.

| **Variable** | **aOR (95% CI*)** | **P-value** |
| --- | --- | --- |
| *Prespecified model including all patients* | | |
| Embolisation | 1.1 (0.4 – 3.2) | 0.85 |
| Eloquent location | 2.0 (0.75 – 5.4) | 0.17 |
| Nidus diameter (per cm) | 0.90 (0.61 – 1.3) | 0.57 |
| Deep venous drainage | 1.3 (0.47 – 3.6) | 0.61 |
| *Sensitivity analysis of only patients with deep venous drainage* | | |
| Embolisation | 4.1 (0.69 – 33.6) | 0.14 |
| Eloquent location | 3.1 (0.69 – 15.7) | 0.15 |
| Nidus diameter (per cm) | 0.73 (0.31 – 1.6) | 0.44 |
| *Exploratory analysis including association with emergency treatment* | | |
| Embolisation | 1.3 (0.42-4.0) | 0.64 |
| Eloquent location | 2.1 (0.78-5.8) | 0.14 |
| Nidus diameter (per cm) | 0.90 (0.60-1.3) | 0.56 |
| Deep venous drainage | 1.4 (0.48-3.8) | 0.56 |
| Emergency surgery | 2.0 (0.56-6.7) | 0.28 |

**Supplementary Table 7:** Binomial logistic regression analyses of association of embolisation with death or dependency (mRS 3-6) adjusted for Spetzler-Martin grade components. aOR Adjusted odds ratio; *95% Confidence interval.

| **Variable** | **aOR (95% CI*)** | **P-value** |
| --- | --- | --- |
| *Prespecified model including all patients* | | |
| Embolisation | 0.54 (0.17 – 1.6) | 0.27 |
| Eloquent location | 1.3 (0.49 – 3.6) | 0.57 |
| Nidus diameter (per cm) | 1.2 (0.87 – 1.7) | 0.25 |
| Deep venous drainage | 1.9 (0.70 – 5.4) | 0.20 |
| *Sensitivity analysis of only patients with deep venous drainage* | | |
| Embolisation | 0.55 (0.09 – 2.9) | 0.49 |
| Eloquent location | 2.7 (0.64 – 11.5) | 0.18 |
| Nidus diameter (per cm) | 1.4 (0.67 – 3.0) | 0.39 |
| *Exploratory analysis including association with emergency treatment* | | |
| Embolisation | 0.81 (0.02-0.28) | 0.73 |
| Eloquent location | 1.6 (0.24-2.7) | 0.41 |
| Nidus diameter (per cm) | 1.3 (0.88-1.8) | 0.21 |
| Deep venous drainage | 2.4 (0.79-7.5) | 0.13 |
| Emergency surgery | 7.7 (2.2-30.4) | 0.0019 |

**Supplementary Table 8:** Binomial logistic regression analyses of association of embolisation with mortality adjusted for Spetzler-Martin grade components. aOR Adjusted odds ratio; *95% Confidence interval.

| **Variable** | **aOR (95% CI*)** | **P-value** |
| --- | --- | --- |
| *Prespecified model including all patients* | | |
| Embolisation | 0.43 (0.019 – 4.1) | 0.50 |
| Eloquent location | 4.6 (0.49 – 101.7) | 0.21 |
| Nidus diameter (per cm) | 1.3 (0.73 – 2.1) | 0.35 |
| Deep venous drainage | 0.52 (0.024 – 4.9) | 0.60 |
| *Sensitivity analysis of only patients with deep venous drainage* | | |
| Embolisation | 8.5x10^7^ (0 – NA) | >0.9 |
| Eloquent location | 7.9x10^8^ (0 – NA) | >0.9 |
| Nidus diameter (per cm) | 4.9 (0.24 – 4856) | 0.50 |
| *Exploratory analysis including association with emergency treatment* | | |
| Embolisation | 2.1 (0.066-75.7) | 0.64 |
| Eloquent location | 7.6 (0.57-243) | 0.15 |
| Nidus diameter (per cm) | 1.3 (0.67-2.5) | 0.38 |
| Deep venous drainage | 0.62 (0.025-8.3) | 0.72 |
| Emergency surgery | 35.7 (2.3-1858) | 0.028 |
